# The impact of surgical excision of the primary tumor in stage IV breast cancer on survival: a meta-analysis

**DOI:** 10.18632/oncotarget.23189

**Published:** 2017-12-13

**Authors:** Shuangshuang Lu, Jiayi Wu, Yan Fang, Wei Wang, Yu Zong, Xiaosong Chen, Ou Huang, Jian-Rong He, Weiguo Chen, Yafen Li, Kunwei Shen, Li Zhu

**Affiliations:** ^1^ Comprehensive Breast Health Center, Ruijin Hospital, Shanghai Jiaotong University School of Medicine, Shanghai 200025, P.R. China

**Keywords:** stage IV breast cancer, surgical excision, primary tumor, survival, meta-analysis

## Abstract

**Introduction:**

Approximately 5% of primary breast cancer patients present *de novo* stage IV breast cancer, for whom systematic therapy is the mainstream treatment. The role of surgical excision of the primary tumor has been controversial due to inconsistent results of relevant studies. Recently, with the reports of some relevant preclinical data, retrospective studies and randomized clinical trials, we've got more evidence to reexamine the issue. Based on those above, a literature review and meta-analysis was performed to determine whether surgery of the primary tumor could improve overall survival in the setting of stage IV breast cancer.

**Materials and Methods:**

A comprehensive search of PubMed, OVID, American Society of Clinical Oncology (ASCO) symposium documents, European Society for Medical Oncology (ESMO) symposium documents and San Antonio Breast Cancer Symposium (SABCS) symposium documents was performed to identify published literature that evaluated survival benefits from excision of the primary tumor in the setting of stage IV breast cancer. Data were extracted in review of appropriate studies by the authors independently. The primary endpoint was overall survival following surgical removal of the primary tumor. Secondary endpoints were the impacts of surgery on progression free survival (PFS) and time to progression (TTP).

**Results:**

Data from 19 retrospective studies showed a pooled hazard ratio of 0.65 (95% confidence interval (95% CI), 0.60-0.71, *P* < 0.01= for overall survival (OS), indicating a 35% reduction in risk of mortality in patients who underwent surgical excision of the primary tumor. Nevertheless, the analysis of 3 randomized clinical trials revealed a pooled hazard ratio of 0.85 (95% CI, 0.59–1.21, *P* = 0.359) for OS in the surgical group. According to the meta-regression, the survival benefit was independent of age, tumor size, site of the metastases, and PR or HER-2 status, acceptance of systematic therapies and radiotherapy and inversely correlated with the ER+ status of the population included.

**Conclusions:**

This is the first meta-analysis that includes both retrospective and prospective studies regarding the impact of surgery of the primary tumor on survival in stage IV breast cancer patients. According to the analytical results, we do not recommend surgery of the primary tumor as routine therapy for stage IV breast cancer. However, for those who are supposed to have long life expectancy, physicians could discuss it with these patients, put forward surgery as a therapy choice and perform the operation under deliberation.

## INTRODUCTION

Breast cancer is the second common cancer in the world, and by far, the most frequent cancer among women with an estimated 1.67 million new cancer cases diagnosed in 2012 (25% of all cancers) [1]. In the United States and Western Europe, approximately 5% of women with primary breast cancer present de novo stage IV breast cancer [2]. The mainstream treatments in such advanced disease are systematic therapies including chemotherapy, endocrine therapy and target therapy. The surgery of the primary tumor is usually applied to obtain local control, alleviate cancer-related symptoms and meet the patients’ desire. However, there are some hypotheses suggesting that removal of the primary tumor would help obtain control of the disease and reduce the distant spread of tumor cells. One hypothesis is the “self-seed” model. In this model, the primary tumor acts as a source of continued seeding of distant metastatic sites, which could “self-seed” and circulate back to the primary tumor, accelerating growth and angiogenesis through cytokine action [3]. The possibility that removal of the primary tumor may restore immuno-competence is also suggested by an investigation using a mouse model, showing that when compared with healthy mice, those with metastatic disease had recovery of antigen-specific antibody responses and T-cell responses to foreign antigens after removal of the primary tumor [4]. Besides, a study by Campbell et al shows both CD4-positive and CD8-positive T-cell subsets producing type 1 (IL-2, IFN-γ or TNF-α) and type 2 (IL-4) cytokines were significantly reduced in patients with breast cancer, and they also observed a correlation between number of micrometastases in the bone marrow and T cell responsiveness [5]. Based on these hypotheses, surgery of the primary tumor is likely to control the disease and further improve the survival of stage IV breast cancer patients.

Besides, “low-burden” stage IV disease is increasingly identified with sensitive imaging modalities such as PET/CT. Together with the application of modern systematic therapies such as trastuzumab, survival of stage IV patients seems to be improving [6]. The removal of the primary tumor has already been shown to improve survival in other metastatic disease such as melanoma [7], renal cell carcinoma [8], colorectal cancer [9] and gastric cancer [10]. Treatments for stage IV breast cancer patients should also be re-examined.

There are already some retrospective studies and randomized clinical trials evaluating survival benefits from excision of the primary tumor in stage IV breast cancer, but the results are ambiguous and the role of surgery remains unclear. These controversies compelled us to perform a systematic review and meta-analysis to further explore it. A meta-regression weighted for age, visceral or bone disease, rate of radiotherapy, systematic therapies offered, tumor size, ER status and HER-2 status was also performed.

## MATERIALS AND METHODS

### Search methods for the identification of eligible studies

A search of PubMed, OVID, ASCO symposium documents, ESMO symposium documents, and SABCS symposium documents was performed using the following keywords in the searching algorithm: Stage IV OR metastatic AND Breast Cancer AND Surgery OR Surgical Excision. We set English language as a restriction. The latest search was done on August 15, 2016. Two authors (Lu S and Wu J) independently examined the title and abstract citations. All relevant texts, tables, and figures were reviewed for data extraction.

### Inclusion and exclusion criteria

A prospective clinical trial or retrospective study of more than 50 patients with stage IV breast cancer was included if it compared the overall survival (OS) between the patients who underwent surgical excision of the primary tumor and patients who didn’t. It was imperative that all patients were staged according to the TNM or AJCC Cancer Staging Manual [11] and the hazard ratios (HRs) for OS should be reported. Type of surgery could be either conservative (lumpectomy or tumorectomy) or demolitive (mastectomy) and could be associated with axillary node dissection (or sentinel node biopsy) or not. Studies were excluded if the survival data were incomplete, or have repeating datasets but less detailed analysis compared with other reports. Reviews, case reports, letters and commentaries were excluded.

### Data extraction and management

Data were extracted by the authors independently using the following items: characteristics of included studies (author’s name, journal, year of publication), baseline characteristics of included patients (sourcing of patients, median age, histology, ER status, PR status, HER-2 status, metastatic sites, number of metastases, nodal status and type of surgery), and the survival data. The Guidelines in the Cochrane Handbook for Systematic Review of Interventions [12] were consulted from the methods to the presentation of results to the discussion of this meta-analysis.

### Statistical analysis

Hazard Ratios (HRs) and their 95% confidential intervals (95% CIs) for OS, PFS, and TTP as benefit of surgery with or without other treatment modalities were retrieved from each study. Meta-analyses of HRs were performed with both fixed-effect and random-effect models. Statistical heterogeneity among the included studies was assessed using Cochran’s *Q* test, and a Χ^2^ test and I^2^ statistic test were used to quantify the inconsistency. The assumption of homogeneity was considered invalid for *P* < 0.1. We took the random-effects model to calculate the pooled HR if the Χ^2^ test for heterogeneity showed *P* < 0.01 or the I^2^ test showed an index of more than 50%, otherwise fixed-effects models were adopted.

Meta-regression analysis was performed to balance the results for specific covariates such as ER status, PR status and HER-2 status, receipt of systematic therapies (including chemotherapy, hormonal therapy and anti-HER-2 therapy) mastectomy rate, margin status, use of radiotherapy (either to the primary tumor or the metastatic sites), performance status, median age, number of metastatic sites (1 vs > 1), and type of metastases (visceral vs bone/soft tissue metastasis), whichever was available.

Finally, potential publication biases were evaluated with Begg’s funnel plots for OS and subsequently with Begg’s tests. A two-tailed *P* value of < 0.05 was considered statistically significant. Results of the meta-analyses were reported as a classical forest plot or data tables. Statistical analyses were performed using STATA version 12.0 and SPSS version 21.0.

## RESULTS

**Figure 1:**
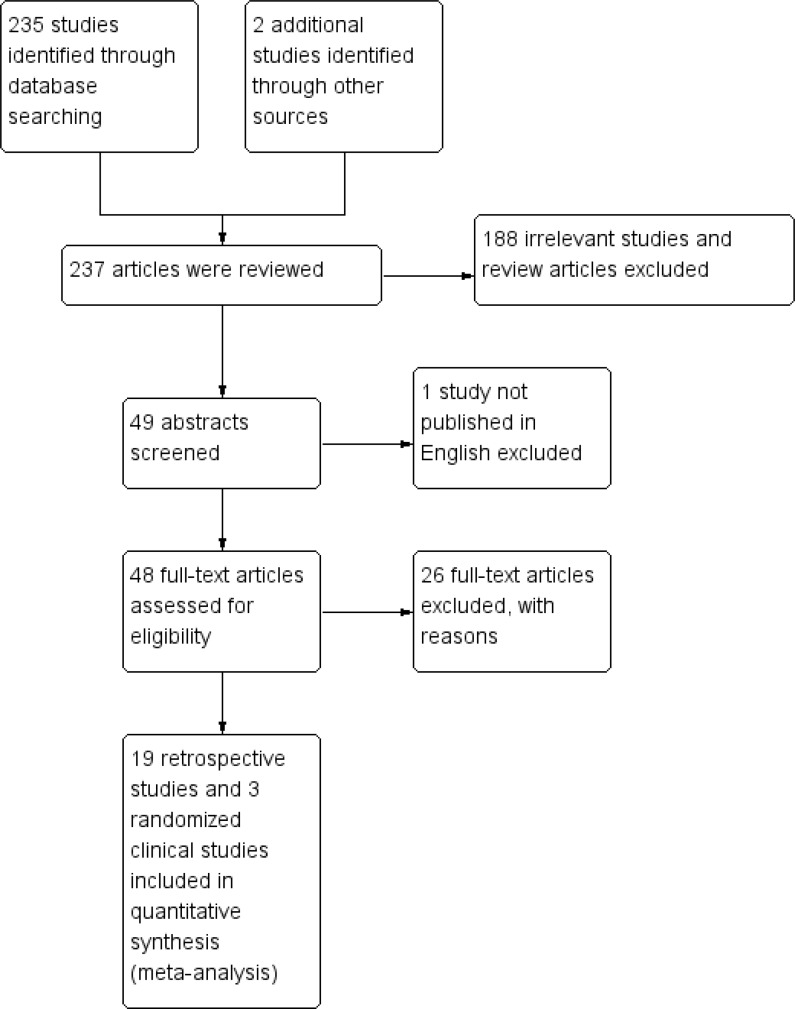
Consort flow of studies selected for meta-analysis comparing surgery versus no surgery of the primary tumor in the setting of stage IV disease

A total of 237 articles were reviewed and 49 articles initially met the inclusion criteria, then 27 articles were excluded due to lack of HRs for OS in the multivariate analysis, not written in English, incomplete data sets or repeated datasets (Figure 1). Finally, 19 retrospective cohort studies [13–31] and 3 randomized clinical trials met the full inclusion criteria and were included in this meta-analysis [32, 33] (Supplementary Table 1).

### Patient and tumor characteristics

A total of 67682 patients were included in the 19 retrospective studies and 46.4% of them were treated with surgery. Seven hundred and fourteen patients were included in the 3 randomized clinical trials with 49.0% undergoing surgery.

Data of surgery type were available in 16 studies; 14122 (62.2%) patients underwent a mastectomy, while 8570 (37.8%) were treated with breast-conserving procedures. Patients undergoing surgery were younger in all studies except the study by Dominici [17]. As for the tumor size, 65.4% of the patients in the surgical group had a tumor that was T2 or smaller compared with 54.0% in the nonsurgical group (*P* < 0.001). In the surgical group, 8.8% patients had low-grade tumors, 32.7% had moderate-grade tumors and 58.5% had high-grade tumors, compared with 6.5%, 36.3% and 57.1% respectively in the non-surgery group. 69.6 % of the surgical group was ER positive compared with 72% of the nonsurgical group (*P* < 0.001). There was no significant difference of PR status (56.0% in surgical group versus 56.5% in nonsurgical group, *P* = 0.473) and HER-2 status (36.9% in surgical group versus 34.4% in nonsurgical group, *P* = 0.427) between the two groups. Furthermore, patients in the surgical group had fewer metastases than their counterparts in the analysis of retrospective studies. (73.7% patients in the surgical group had metastasis in only one location versus 55.6% in the nonsurgical group (*P* < 0.001). Fewer patients in the surgical group had visceral metastasis (52.8% versus 56.7%, *P* = 0.023) and the proportion of patients with bone metastasis is higher in the surgery group than in the non-surgery group (44.5% versus 40.8%, *P* = 0.034).

In the randomized clinical trials, patients undergoing surgery had larger tumors (3.8 cm versus 3.6 cm, *P* = 0.01) and were more likely to have single organ metastasis (*P* = 0.001) in the TBCRC 013 clinical trial [34], whereas baseline demographic and disease characteristics were well balanced between groups in the other two clinical trials [32, 33, 35].

### Primary endpoint (overall survival)

**Figure 2:**
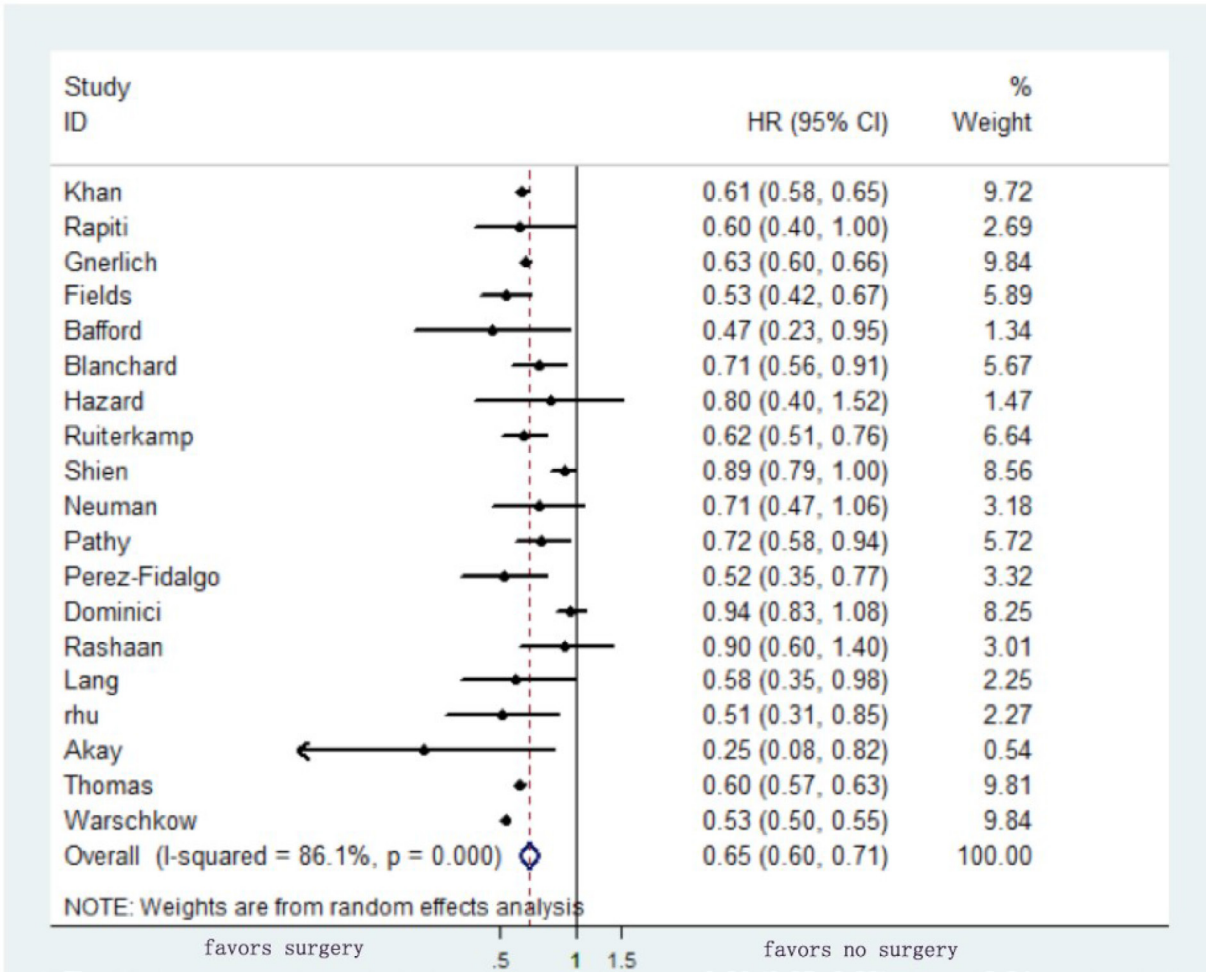
Pooled analysis of hazard ratio for overall survival of surgery versus no surgery of the primary tumor in patients with stage IV breast cancer

**Figure 3:**
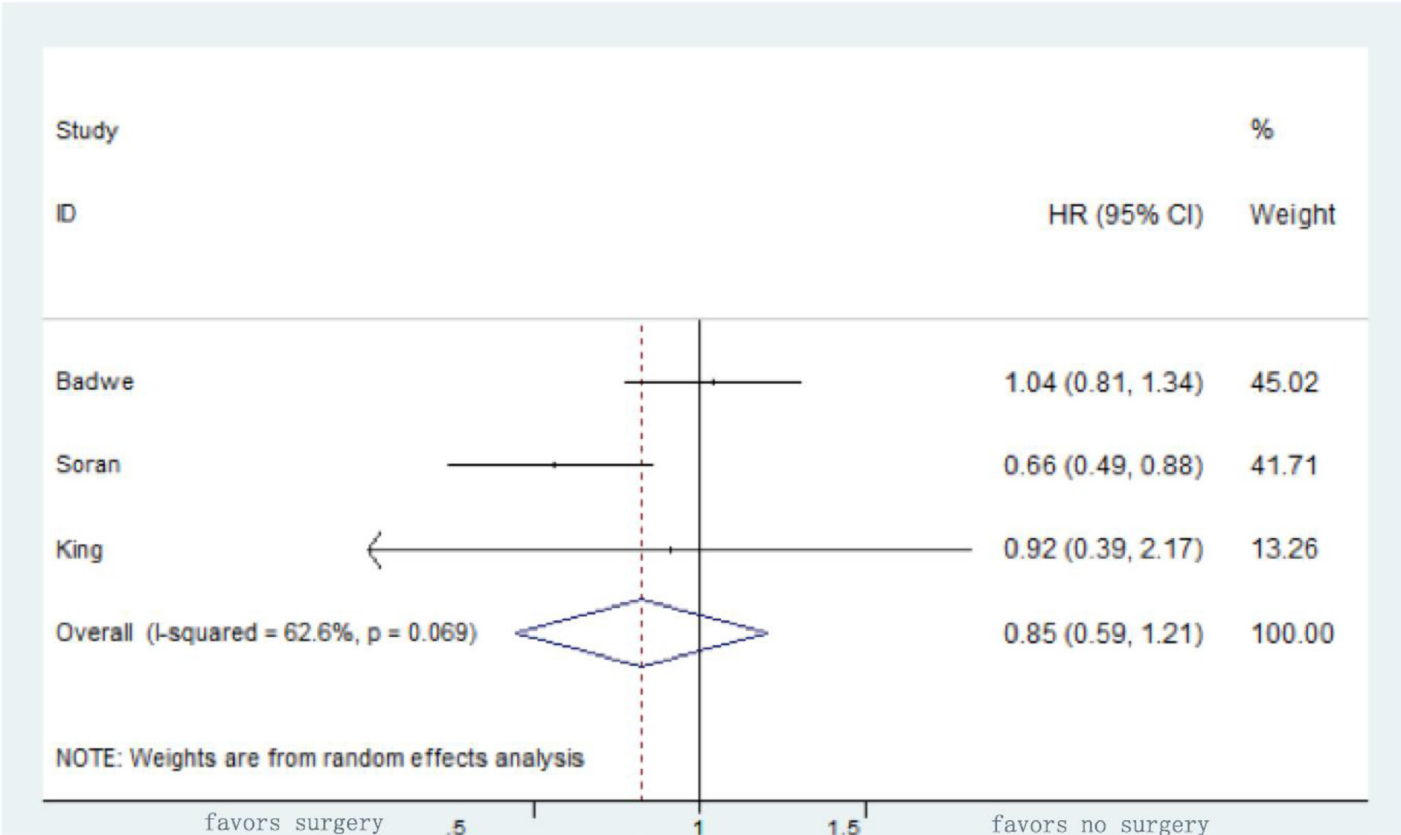
Forrest box-plot of survival meta-analysis of the randomized clinical trials comparing surgical excision of the primary tumor with no surgery for stage IV breast cancer patients

The HRs for OS and standard errors for the estimated HRs were reported or extrapolated from all studies. A random-effects model was adapted to calculate the pooled HR of surgery for OS in the retrospective studies, which was 0.65 with a 95% CI of 0.6-0.71, confirming the assumption that surgery is beneficial in terms of reducing the risk of mortality by 35% (Figure 2). As for the randomized clinical trials, the pooled HR for OS was 0.85 with a 95% CI of 0.59-1.21 (*P* = 0.359) (Figure 3).

**Figure 4:**
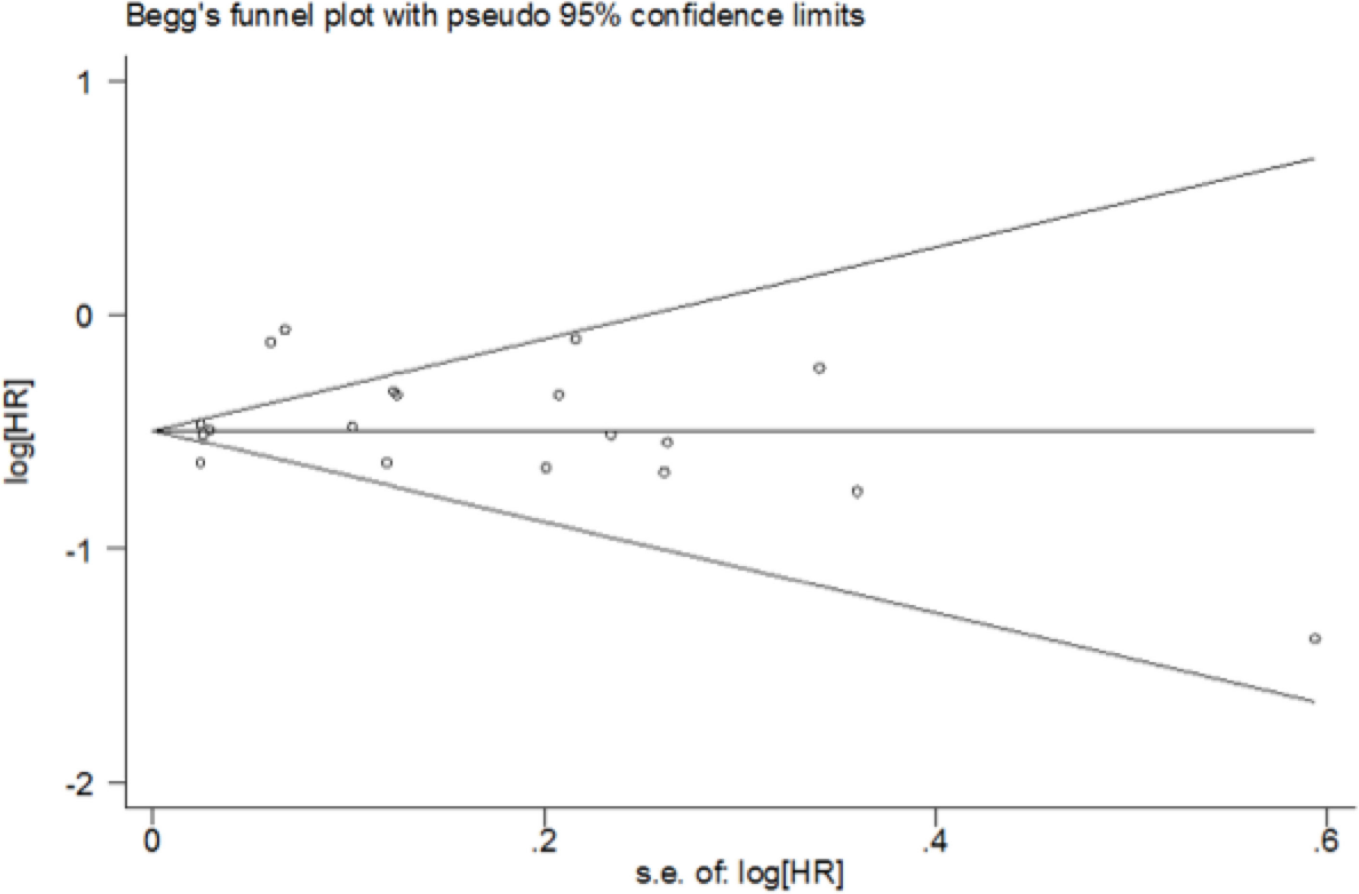
Funnel plot for overall survival meta-analysis All previous studies, with the exception of the studies by Dominici, Rashaan, and Shien *et al*, were within the 95% confidence intervals and were relatively symmetrically placed.

The funnel plot for risk of bias in OS in the retrospective studies revealed that all studies, with the exceptions of Dominici, Rashaan and Shien et al [17], fell within the 95% CI, and were relatively symmetrically distributed (Figure 4). Evidence of publication bias was not revealed in the present analysis (Begg’s test, *P* = 0.363).

### Secondary endpoints

Only three trials among those analyzed reported data about PFS/TTP. Hazard et al. reported a significant HR of 0.493 (for time to first progression for resected patients in multivariate analysis, *P* = 0.015). Lang et al. reported a benefit for surgery in PFS with an HR of 0.40 (*P* < 0.0001) from multivariate analysis. Fields et al. suggested in the multivariate analysis that there was no significant difference between the time to metastatic progression in surgical and non-surgical group (HR = 1.0, *P* = 0.378). A formal meta-analysis was not possible due to the limited data available.

### Meta-regression analysis

A meta-regression was performed to explore whether the HR for OS is affected by some other explanatory variables mentioned above. An inverse correlation of HR for OS and ER positive status was discovered (*P* = 0.03). A conversely positive association between OS and surgery of the primary tumor remained significantly independent of number of sites of disease, rate of bone or visceral metastasis, type of surgery, margin status, and median age.

## DISCUSSION

The standard treatments for patients with stage IV breast cancer are systematic therapies, and surgical excision of the primary tumor is usually applied to palliate symptoms rather than to cure. All of the previous meta-analysis and most of the regarding retrospective studies suggest that it is beneficial to perform an operation of the primary tumor for stage IV breast cancer patients. And with the emergency of the “self-seed” and “immuno-competence restore” model, the value of surgery of the primary tumor in stage IV breast cancer becomes the stuff of increasingly fierce debate.

This is the first meta-analysis studying the impact of surgery of the primary tumor on the survival of stage IV breast cancer patients that included both retrospective and prospective studies. Random-effects models were adopted to calculate the pooled HR of surgery for OS, which was 0.65 with a 95% CI of 0.6-0.71 in the retrospective studies, indicating a 35% reduction in risk of mortality for patients undergoing surgery, and 0.85 with a 95% CI of 0.59-1.21 in the randomized clinical trials, showing no statistically significant difference between the two groups.

Although the statistical result of the retrospective studies strongly suggests that surgical excision of the primary tumor in patients with stage IV breast cancer is beneficial in terms of survival, our analysis shows a large extent of selection bias in these studies. In those retrospective studies, women who received surgery tended to be younger, have smaller tumors, fewer comorbidities, lower burden of metastatic disease, less likely to have visceral metastases, and more likely to have access to better care. Differences between studies regarding inclusion criteria and study designs should not be neglected either. Furthermore, the retrospective studies covered the period 1962-2011 but we’ve made great progress in systematic therapy these years. When taking the results of the randomized clinical trials and the levels of evidence into account, we do not recommend surgery of the primary tumor as routine therapy for stage IV breast cancer patients.

Despite issues above, patients with good profile may gain longer survival, and they are liable to greater risk of losing local control and reduced quality of life over time. The study by Shien et al [30] also showed that patients with favorable profiles seemed to have the most benefit from surgery. What’s more, according to our analysis, ER+ status is a positive predictor for OS of patients with stage IV breast cancer. Therefore, for these patients, especially hormonal receptor positive patients, surgery of the primary tumor may be of benefit in preventing local progression and even prolonging their survival. In clinical practice, physicians could discuss it with this kind of patients, put forward surgery as a therapy choice and perform the operation under deliberation.

There are also some limitations to this meta-analysis and its conclusions. This meta-analysis includes only three randomized clinical trials and there are also certain disparities in the design of these clinical trials. For example, only patients who had good response to the previous systematic therapy could get surgery in the TBCRC 013 trial [34], which weakens the stringency of this conclusion. And the final results of two of them are not fully exposed.

With the evolvement of adjuvant therapies and the paradigm shift of viewing stage IV diseases as chronic disease to be managed rather than a terminal event, the role of surgery will still be evolving, and we’re looking forward to the disclosure of the other randomized clinical trials to help us to see it clearly.

## SUPPLEMENTARY MATERIALS TABLE




