# Supplementary material for: The impact of surgical excision of the primary tumor in stage IV breast cancer on survival: a meta-analysis
Source: Oncotarget. 2017 Dec 13;9(14):11816–23. doi: 10.18632/oncotarget.23189 (PMC5837759; doi:10.18632/oncotarget.23189)
Supplement: Supplementary file 1 [file oncotarget-09-11816-s001.pdf]

The impact of surgical excision of the primary tumor in stage iv breast cancer on survival: a meta-analysis

SUPPLEMENTARY MATERIALS

Supplementary Table 1: Available data on tumor and metastatic characteristics of the patients undergoing/not undergoing surgical excision of the primary tumor Studies included in this meta-analysis. See_Supplementary_Table 1
